# Supplementary material for: Plasma Immune Proteins and Circulating Tumor DNA Predict the Clinical Outcome for Non-Small-Cell Lung Cancer Treated with an Immune Checkpoint Inhibitor
Source: Cancers (Basel). 2023 Nov 29;15(23):5628. doi: 10.3390/cancers15235628 (PMC10705682; doi:10.3390/cancers15235628)
Supplement: Supplementary file 1 [file cancers-15-05628-s001.zip › Supplementary Table S1.pdf]

Supplementary Table S1: Baseline characteristics

Characteristics	Patient cohort (n = 42)	High FASLG/ICOSLG expression (n=28)	Low FASLG/ICOSLG expression (n=14)	*p* value
**Age at treatment initiation (years)**				
Median (range)	68 (51-85)	68 (51-85)	68 (56-81)	0.87
**Sex, n (%)**				
Female	22 (52.38)	15 (53.57)	7 (50.00)	>0.99
Male	20 (47.62)	13 (46.43)	7 (50.00)	
**Histopathology, n (%)**				
Adenocarcinoma	34 (80.95)	22 (78.57)	12 (85.71)	>0.99
Squamous cell carcinoma	7 (16.67)	5 (17.86)	2 (14.29)	
Not otherwise specified[†]	1 (2.38)	1 (3.57)	0 (0.00)	
**Metastasis stage, n (%)**				
No metastasis	15 (35.71)	10 (35.71)	5 (35.71)	0.23
Pulmonary contralateral metastasis or pleural/pericardial effusion	9 (21.43)	4 (14.29)	5 (35.71)	
Extrapulmonary metastasis	18 (42.86)	14 (50.00)	4 (28.57)	
**Treatment line, n (%)**				
First	33 (78.57)	23 (82.14)	10 (71.43)	0.45
Second	9 (21.43)	5 (17.86)	4 (28.57)	
**Smoking status, n (%)**				
Active	11 (26.19)	5 (17.86)	6 (42.86)	0.14
Former	30 (71.43)	22 (78.57)	8 (57.14)	
Never[†]	1 (2.38)	1 (3.57)	0 (0.00)	
**Performance status, n (%)**				
0	14 (33.33)	11 (39.29)	3 (21.43)	0.19
1	25 (59.52)	14 (50.00)	11 (78.57)	
2[†]	3 (7.14)	3 (10.71)	0 (0.00)	
**Comorbidity score, n (%)**				
0-1	32 (76.19)	21 (75.00)	11 (78.57)	>0.99
2-5	10 (23.81)	7 (25.00)	3 (21.43)	
**PD-L1 TPS, n (%)**				
0-49%	7 (16.67)	4 (14.29)	3 (21.43)	0.67
50-100%	35 (83.33)	24 (85.71)	11 (78.57)	
**Best overall response, n (%)**				
PR	19 (45.23)	14 (50.00)	5 (35.71)	0.75
SD	11 (26.19)	7 (25.00)	4 (28.57)	
PD	10 (23.81)	6 (21.43)	4 (28.57)	
Unknown[†,a]	2 (4.76)	1 (3.57)	1 (7.14)	

**ctDNA, n (%)‡**				>0.99
Mutation negative	13 (30.95)	9 (32.14)	4 (28.57)	
Mutation positive	28 (66.67)	18 (64.29)	10 (71.43)	

ctDNA, circulating tumor DNA; PD, progressive disease; PD-L1, programmed cell death ligand 1; PR, partial response; SD, stable disease; TPS, tumor proportion score. †, subgroup was not included in the statistical analysis, since the number of observations did not meet the requirement for χ^2-test. ‡, one patient in the low FASLG/ICOSLG expression group did not have available plasma for ctDNA testing. [a], patients with no available radiological assessment of response.
