# Supplementary material for: Plasma Immune Proteins and Circulating Tumor DNA Predict the Clinical Outcome for Non-Small-Cell Lung Cancer Treated with an Immune Checkpoint Inhibitor
Source: Cancers (Basel). 2023 Nov 29;15(23):5628. doi: 10.3390/cancers15235628 (PMC10705682; doi:10.3390/cancers15235628)
Supplement: Supplementary file 1 [file cancers-15-05628-s001.zip › Supplementary Table S2.pdf]

Supplementary Table S2: Univariate and multivariate Cox regression analyses

Variable	Univariate		Multivariate	
	p value	HR (95% CI)	*p* value	HR (95% CI)
**PFS**				
Age [cont.]	0.056	1.04 (1.00-1.09)		
Histology [sq. cell]	0.023	2.69 (1.06-5.95)	0.021	2.94 (1.10-7.07)
Sex [F]	0.71	0.89 (0.47-1.70)		
Smoking [active]	0.77	1.12 (0.51-2.24)		
PD-L1 TPS [>50%]	0.26	0.62 (0.28-1.56)		
Treatment line [2. line]	0.22	1.61 (0.71-3.32)		
PS				
[1]	0.095	1.85 (0.92-3.96)		
[2]	0.50	1.55 (0.35-5.01)		
Co-morbidity [cont.]	0.49	1.08 (0.85-1.32)		
$KRAS_{tissue}$ [mutated]	0.17	0.51 (0.17-1.21)		
$KRAS_{ctDNA}$ [mutated]	0.25	0.67 (0.33-1.30)		
$TP53_{ctDNA}$ [mutated]	0.30	0.71 (0.37-1.35)		
ICOSLG [above median]	0.051	0.51 (0.26-1.00)	0.042	0.47 (0.23-0.96)
FASLG				
[above median]	0.16	0.63 (0.33-1.20)		
[upper quartile]	0.009	0.34 (0.14-0.73)	0.074	0.47 (0.19-1.03)
**OS**				
Age [cont.]	0.45	1.02 (0.98-1.06)		
Histology [Sq. cell]	0.027	2.65 (1.04-5.99)	0.057	2.35 (0.91-5.45)
Sex [F]	0.78	0.91 (0.46-1.80)		
Smoking [Active]	0.77	1.12 (0.49-2.33)		
PD-L1 TPS [>50%]	0.13	0.52 (0.24-1.32)		
Treatment line [2. Line]	0.063	2.07 (0.91-4.30)		
PS				
[1]	0.055	2.13 (1.02-4.86)		
[2]	0.39	0.40 (0.02-2.15)		
Co-morbidity score [cont.]	0.37	1.10 (0.88-1.35)		
$KRAS_{tissue}$ [mutated]	0.053	0.31 (0.07-0.87)		

KRAS$_{ctDNA}$ [mutated]	0.080	0.51 (0.23-1.05)		
TP53$_{ctDNA}$ [mutated]	0.62	1.19 (0.60-2.35)		
ICOSLG [above median]	0.025	0.44 (0.21-0.89)	0.046	0.47 (0.22-0.98)
FASLG				
[above median]	0.19	0.63 (0.32-1.26)		
[upper quartile]	0.010	0.30 (0.11-0.70)	0.062	0.41 (0.15-0.99)

Variable tested is shown in brackets. Baseline characteristics with *p* values below 0.05 and significant protein expressions from Kaplan-Meier analyses were included in the multivariate Cox regression analysis. CI, confidence interval; cont., continuous variable; ctDNA, circulating tumor DNA; F, female; HR, hazard ratio; PD-L1, programmed cell death ligand 1; PFS, progression-free survival; PS; performance status; sq. cell; squamous cell carcinoma; TPS, tumor proportion score.
