# Supplementary material for: Plasma Immune Proteins and Circulating Tumor DNA Predict the Clinical Outcome for Non-Small-Cell Lung Cancer Treated with an Immune Checkpoint Inhibitor
Source: Cancers (Basel). 2023 Nov 29;15(23):5628. doi: 10.3390/cancers15235628 (PMC10705682; doi:10.3390/cancers15235628)
Supplement: Supplementary file 1 [file cancers-15-05628-s001.zip › Supplementary Table S3.pdf]

Supplementary Table S3: Proteins in the Olink Target 96 Immuno-Oncology Panel

Protein abbreviation	Full protein name	UniProt ID
ADA	Adenosine deaminase	P00813
ADGRG1	Adhesion G-protein coupled receptor G1	Q9Y653
ANGPT1	Angiopoietin-1	Q15389
ANGPT2	Angiopoietin-2	O15123
ARG1	Arginase-1	P05089
CAIX	Carbonic anhydrase 9	Q99616
CASP-8	Caspase-8	Q14790
CCL17	C-C motif chemokine 17	Q92583
CCL19	C-C motif chemokine 19	Q99731
CCL20	C-C motif chemokine 20	P78556
CCL23	C-C motif chemokine 23	P55773
CCL3	C-C motif chemokine 3	P10147
CCL4	C-C motif chemokine 4	P13236
CD244	Natural killer cell receptor 2B4	Q9BZW8
CD27	CD27 antigen	P26842
CD28	T cell-specific surface glycoprotein CD28	P10747
CD4	T cell surface glycoprotein CD4	P01730
CD40	CD40L receptor	P25942
CD40-L	CD40 ligand	P29965
CD5	T cell surface glycoprotein CD5	P06127
CD70	CD70 antigen	P32970
CD83	CD83 antigen	Q01151
CD8A	T cell surface glycoprotein CD8 alpha chain	P01732
CRTAM	Cytotoxic and regulatory T cell molecule	O95727
CSF-1	Macrophage colony-stimulating factor 1	P09603
CX3CL1	Fractalkine	P78423
CXCL1	C-X-C-motif chemokine 1	P09341
CXCL10	C-X-C-motif chemokine 10	P02778
CXCL11	C-X-C-motif chemokine 11	O14625
CXCL12	Stromal cell-derived factor 1	P48061
CXCL13	C-X-C-motif chemokine 13	O43927
CXCL5	C-X-C-motif chemokine 5	P42830
CXCL9	C-X-C-motif chemokine 9	Q07325
DCN	Decorin	P07585
EGF	Pro-epidermal growth factor	P01133
FASLG	Fas ligand, Tumor necrosis factor ligand superfamily member 6	P48023
FGF2	Fibroblast growth factor 2	P09038
Gal-1	Galectin-1	P09382
Gal-9	Galectin-9	O00182

GZMA	Granzyme A	P12544
GZMB	Granzyme B	P10144
GZMH	Granzyme H	P20718
HGF	Hepatocyte growth factor	P14210
HO-1	Heme oxygenase 1	P09601
ICOSLG	Inducible T cell costimulator ligand	O75144
IFN-gamma	Interferon gamma	P01579
IL-1 alpha	Interleukin-1 alpha	P01583
IL10	Interleukin-10	P22301
IL12	Interleukin-12	P29459, P29460
IL12RB1	Interleukin-12 receptor subunit beta-1	P42701
IL13	Interleukin-13	P36225
IL15	Interleukin-15	P40933
IL18	Interleukin-18	Q14116
IL2	Interleukin-2	P60568
IL33	Interleukin-33	O95760
IL4	Interleukin-4	P05112
IL5	Interleukin-5	P05113
IL6	Interleukin-6	P05231
IL7	Interleukin-7	P13232
IL8	Interleukin-8	P10145
KIR3DL1	Killer cell immunoglobulin-like receptor 3 DL1	P43629
KLRD1	Natural killer cells antigen CD94	Q13241
LAG3	Lymphocyte activation gene 3 protein	P18627
LAMP3	Lysosome-associated membrane glycoprotein 3	Q9UQV4
LAP TGF-beta-1	Latency-associated peptide transforming growth factor beta-1	P01137
MCP-1	C-C motif chemokine 2	P13500
MCP-2	C-C motif chemokine 9	P80075
MCP-3	C-C motif chemokine 7	P80098
MCP-4	C-C motif chemokine 13	Q99616
MIC-A/B	MHS class I polypeptide-related sequence A/B	Q29983, Q29980
MMP12	Matrix metalloproteinase 12	P39900
MMP7	Matrix metalloproteinase 7	P09237
MUC-16	Mucin-16	Q8WXI7
NCR1	Natural cytotoxicity triggering receptor	O76036
NOS3	Nitric oxide synthase, endotheliaL	P29474
PDCD1	Programmed cell death protein 1	Q15116
PDGF subunit B	Platelet-derived growth factor subunit B	P01127
PD-L1	Programmed cell death 1 ligand 1	Q9NZQ7
PD-L2	Programmed cell death 1 ligand 2	Q9BQ51
PGF	Placenta growth factor	P49763
PTN	Pleiotrophin	P21246
TIE2	Angiopoietin-1 receptor	Q02763

TNF	Tumor necrosis factor	P01375
TNFRSF12A	Tumor necrosis factor receptor superfamily member 12A	Q9NP84
TNFRSF21	Tumor necrosis factor receptor superfamily member 21	O75509
TNFRSF4	Tumor necrosis factor receptor superfamily member 4	P43489
TNFRSF9	Tumor necrosis factor receptor superfamily member 9	Q07011
TNFSF14	Tumor necrosis factor ligand superfamily member 14	O43557
TRAIL	TNF-related apoptosis-inducing ligand	P50591
TWEAK	Tumor necrosis factor ligand superfamily member 12	O43508
VEGFA	Vascular endothelial growth factor A	P15692
VEGFR-2	Vascular endothelial growth factor receptor 2	P35968
