# Supplementary figures and images for: Plasma Immune Proteins and Circulating Tumor DNA Predict the Clinical Outcome for Non-Small-Cell Lung Cancer Treated with an Immune Checkpoint Inhibitor
Source: Cancers (Basel). 2023 Nov 29;15(23):5628. doi: 10.3390/cancers15235628 (PMC10705682; doi:10.3390/cancers15235628)

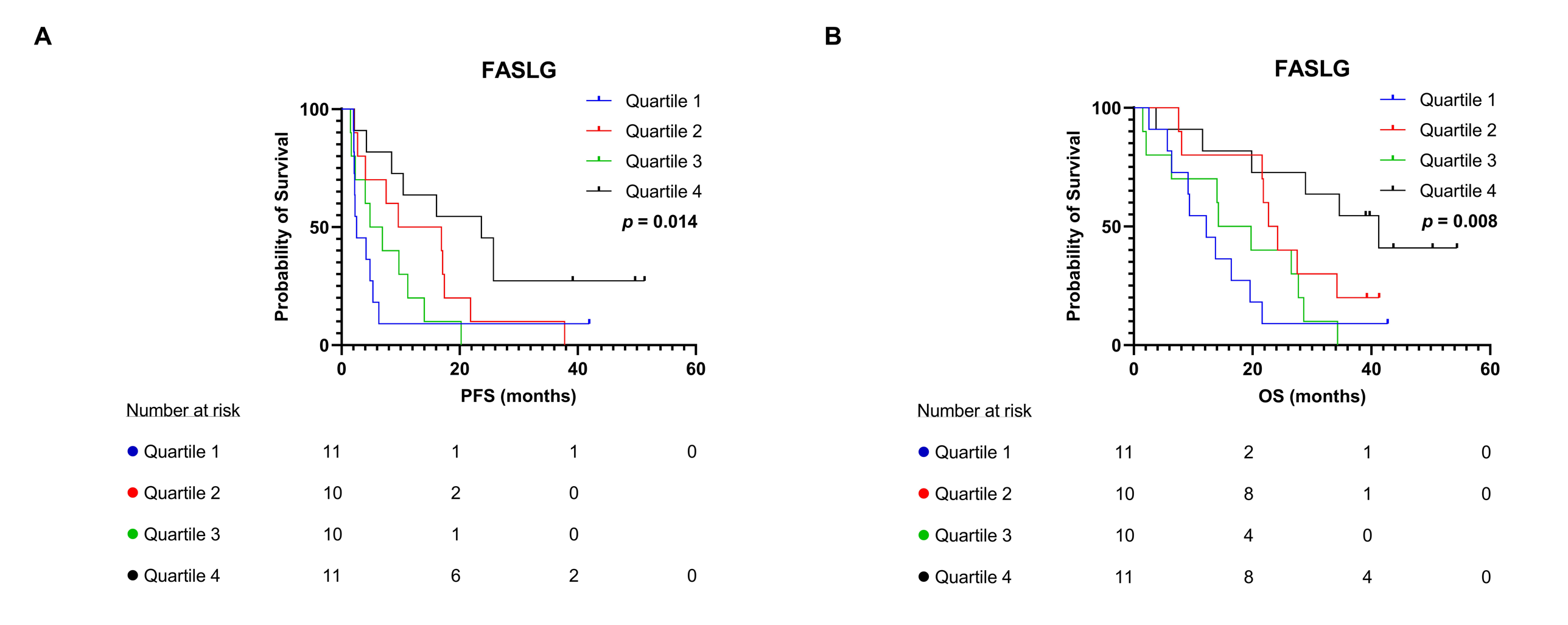

Supplement: Supplementary file 1 [file cancers-15-05628-s001.zip › Supplementary Figure S2.jpg]

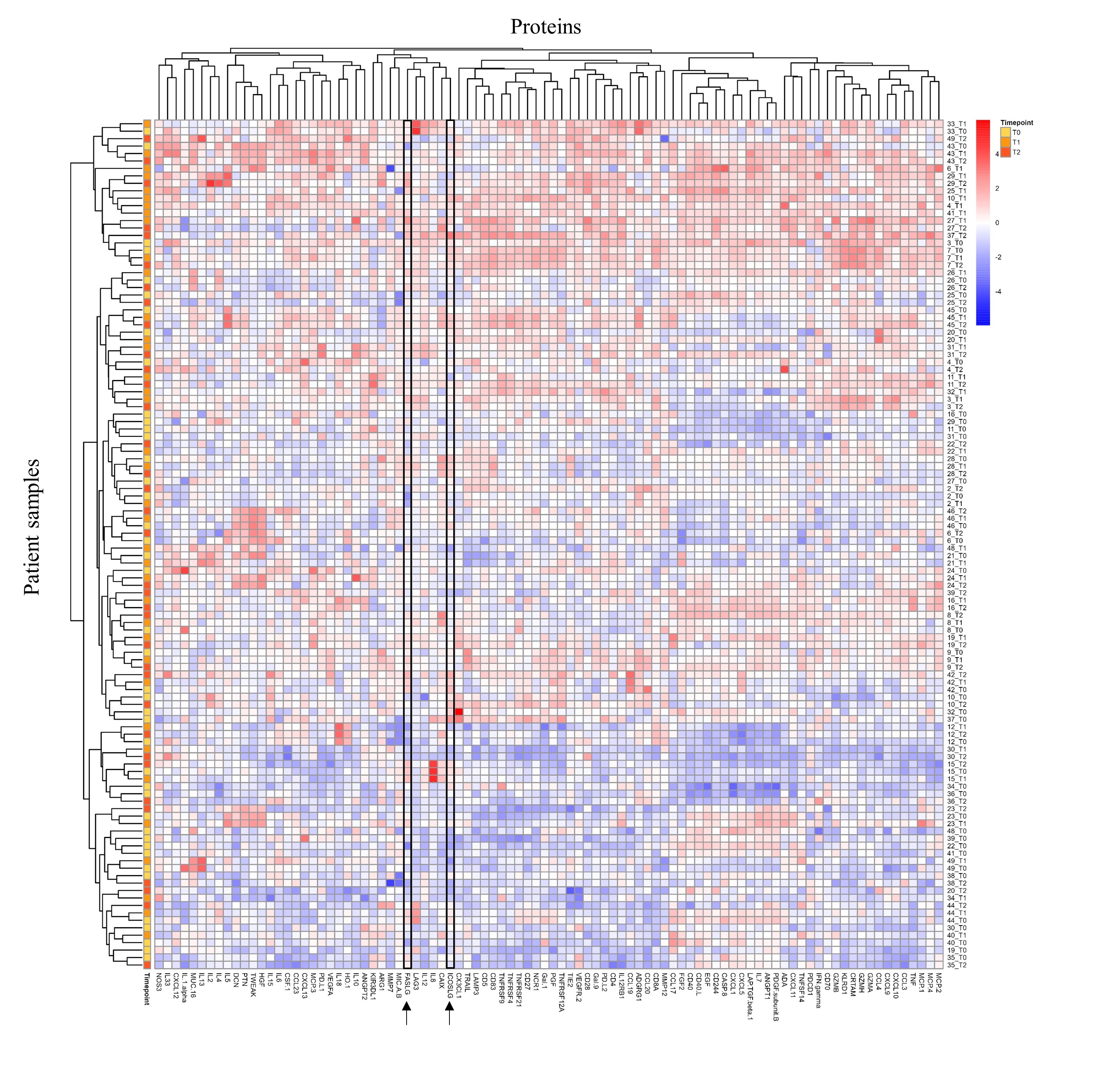

Supplement: Supplementary file 1 [file cancers-15-05628-s001.zip › Supplementary Figure S3.jpg]

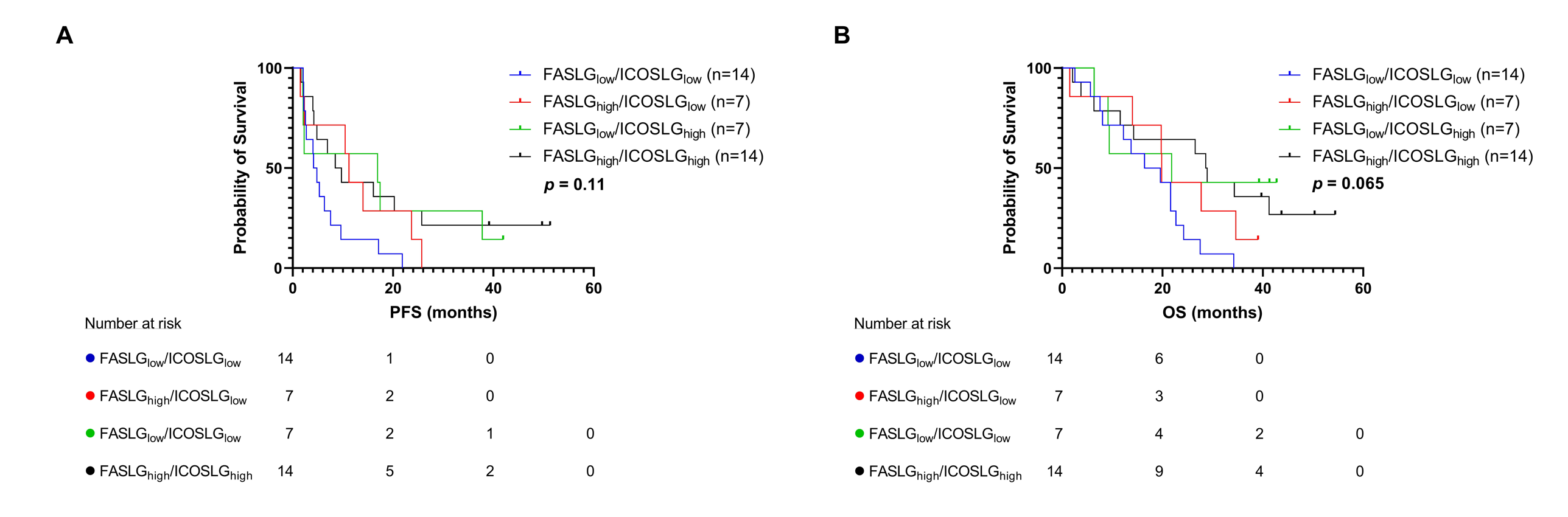

Supplement: Supplementary file 1 [file cancers-15-05628-s001.zip › Supplementary Figure S4.jpg]

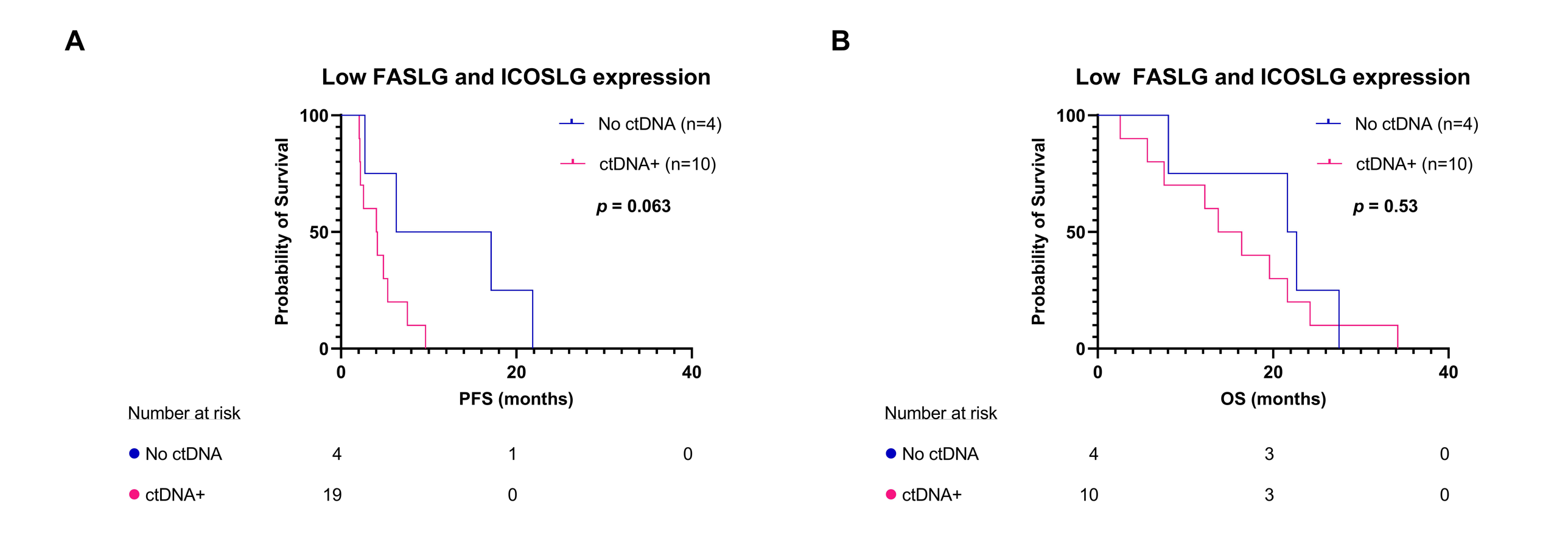

Supplement: Supplementary file 1 [file cancers-15-05628-s001.zip › Supplementary Figure S5.jpg]
